# Bouveret's Syndrome: Literature Review

**DOI:** 10.7759/cureus.2299

**Published:** 2018-03-10

**Authors:** Fady G. Haddad, Wissam Mansour, Liliane Deeb

**Affiliations:** 1 Department of Gastroenterology and Hepatology, Staten Island University Hospital, New York; 2 Department of Pulmonary Critical Care, Staten Island University Hospital, Northwell Health

**Keywords:** bouveret syndrome, gastric outlet obstruction, gallstones

## Abstract

It was in 1896 that Bouveret’s syndrome acquired its name after the French physician Leon Bouveret, who published two case reports in Revue de Medecin. Bouveret’s syndrome describes gastric outlet obstruction secondary to an impacted gallstone. The gallstone reaches the small bowel through a bilioenteric fistula as a consequence of chronic inflammation and adherence between the biliary system and the bowels which increase the intraluminal pressure and leads to secondary wall ischemia and wall perforation with gallstone passage into the bowel. Bouveret’s syndrome’s prevalence is highest among elderly women. Despite the rarity of Bouveret’s syndrome, it can cause notable morbidity and mortality rates. We underwent a review of literature about Bouveret syndrome to increase awareness of its occurrence and potentially life-threatening complications.

## Introduction and background

It was in 1770 that the first case of duodenal obstruction by a gallstone was described by Beaussier. In 1841 Bonnet reported two similar cases by autopsy, but it was not till 1896 that Bouveret’s syndrome acquired its name after the French physician Leon Bouveret, who published two case reports in Revue de Medecin [1]. Bouveret’s syndrome describes gastric outlet obstruction secondary to an impacted gallstone that reached the small bowel through a bilioenteric fistula. The formation of the fistula is a consequence of the chronic inflammation and adherence between the biliary system and the bowels, increasing the intraluminal pressure and causing wall ischemia and wall perforation with gallstone passage into the bowel [2,3].

Bouveret’s syndrome represents 2–3% of all gallstone related obstructions in the gastrointestinal tract, which by itself constitutes 1–4% of all small bowel obstructions, thus the rarity of this syndrome. This can be explained by the fact that only 0.3–5% of gallstones develop fistulas, and that most stones are relatively small and pass either uneventfully or with terminal ileum impaction. Bouveret’s syndrome’s prevalence is highest among elderly women, with a median age at presentation of 74 years and a female to male ratio of 1.9, especially those with gallstones larger than 2.5 cm and postsurgical altered GI anatomy. Despite the rarity of Bouveret’s syndrome, it can cause notable morbidity and mortality rates. The critical association between the rarity and severity of this syndrome should be an incentive for spreading more awareness of its occurrence [2,3].

## Review

By the age of 75, approximately 35% of women and 20% of men have developed gallstones, thus the frequency of this diagnosis. The majority of these patients do well, but complications occur in around 6% of them. That further pushes towards an earlier diagnosis, for the affected population is of an advanced age, with frequent associated comorbidities, which increase the morbidity and mortality risks. Due to improved diagnostic techniques and more restrictive surgical approaches, the mortality rate of Bouveret’s syndrome decreased from 30% to around 12% [2-4].

Bouveret’s syndrome usually presents with non-specific symptoms, most commonly a triad of epigastric pain, nausea, and vomiting. It could also present with abdominal pain, distension, upper gastrointestinal bleeding, fever, weight loss, and anorexia. Physical exam usually shows abdominal tenderness, distension, and dehydration. Differential diagnosis of gastric outlet obstruction in elderly people is subdivided into three groups: inflammatory, malignant, and congenital. Inflammatory causes include peptic ulcer disease, erosive gastritis, and Crohn’s disease. Malignancies include gastric antral carcinoma, duodenal carcinoma, pancreatic carcinoma, ampullary carcinoma, and cholangiocarcinoma, while duodenal web is an example of congenital causes. Variants of this syndrome have been described in the literature, including pyloric obstruction by a gallstone passed through a cholecystogastric fistula, and external compression by a stone located outside the duodenum [2-4].

The diagnosis of Bouveret’s syndrome relies on the clinical presentation and the imaging studies. The initial step of the diagnosis is usually an abdominal plain X-ray, which is diagnostic in only 21% of cases. Abdominal ultrasound confirms Bouveret’s syndrome if it reveals pneumobilia and ectopic location of the gallstone, but excessive intestinal gas and difficulties in locating the gallstone are its limitations. In most cases, computed tomography (CT) scan is needed for the diagnosis, and to view its elaborated evaluation of the fistulas, gallstones and inflammatory findings, its 93% sensitivity, 100% specificity, and 99% accuracy. It is also the best imaging technique used to search for Rigler’s triad that is specific to gallstone ileus, consisting of small bowel obstruction, pneumobilia, and ectopic gallstone. Plain abdominal films reveal Rigler’s triad in 14.8 to 21% of cases, compared to 11.1% for ultrasound and 77.8% for CT scan. Isoattenuation of 15–25% of gallstones on CT scan is a limiting factor, thus the need of additional imaging techniques [2-6].

Magnetic resonance cholangiopancreatography (MRCP) is one of those techniques, providing specific details about fistulas and concrements, delineating clearly fluid from calculi, with the limitation that concrements and air are hardly differentiated. More invasive diagnostic techniques include esophagogastroduodenoscopy (EGD) and surgery, both of which have diagnostic and therapeutic potentials. The first diagnosis by gastroscopy of a pyloric obstruction due to gallstones goes back to 1976. An EGD revealing a dilated stomach, duodenal ulcer with inflammation and edema, cholecystoduodenal fistula, hard non-fleshy mass at the obstruction is helpful in establishing the diagnosis of Bouveret’s syndrome, with a minor possibility of endoscopic removal of the stone. However, obstruction without evidence of stone or fistula is identified in 31% of cases, mainly because the gallstone is compressing the lumen and only partially visualized through the wall. Surgery is needed in 20–40% of cases for the final diagnosis to be made [2-6].

Diagnosis in many cases is challenging and endoscopy is of no benefit. Surgery serves as a diagnostic and therapeutic modality par excellence. The therapeutic approach to Bouveret’s syndrome should always start, as per many authors, by an endoscopic or a percutaneous approach, view the advanced age and medical comorbidities of the affected population, whereas surgery is the mainstay of treatment for gallstone ileus. The endoscopic and percutaneous methods include mechanical lithotripsy, laser lithotripsy, extracorporeal shock wave lithotripsy, and intracorporeal electrohydraulic lithotripsy. Success of these approaches relies on the size of the gallstone, since most of the stones implied in Bouveret’s syndrome are relatively large measuring >2.5 cm. Direct endoscopic removal then increases the risk of stone impaction in the esophagus while fragmentation increases the risk of distal gallstone ileus. Other risks include intestinal wall hemorrhage or perforation. That tends to explain the pursuit of surgery in around 91% of patients for definitive treatment. Note that 42% of surgical patients have already failed an endoscopic trial for gallstone removal [2-6].

The two main surgical approaches are enterolithotomy and gastrotomy, with resection of irreversibly damaged parts of the small bowel. Simultaneous cholecystectomy and fistula repair are still a debate, as supporters of this approach, entitled "one stage surgery", recommend it for the prevention of subsequent biliary complications including cholecystitis and cholangitis, in addition to prevention of occurrence of gallbladder carcinoma. While supporters of the two-stage surgery, consisting of stone removal with subsequent cholecystectomy and fistula repair, pretend the spontaneous closure of the fistula in case of a patent cystic duct and the absence of residual stones in the gallbladder. The rest of the bowel should be examined during surgery to exclude other stones. In the absence of affirming data, the best approach is the one tailored to each patient, with the consideration of his medical condition, age, comorbidities, life expectancy, and operator experience [2-6].

## Conclusions

Bouveret’s syndrome is a rare cause of gastric outlet obstruction by large gallstones that reach the digestive tract through a cholecystoduodenal fistula. Symptoms are non-specific and the typical radiographic triad of intestinal obstruction, ectopic gallstone, and pneumobilia is not uniformly present, and best visualized by CT scan. Therapeutic approach includes three options: a rarely successful endoscopic approach, a technically difficult mechanical and intracorporeal lithotripsy, and an almost always needed surgical approach.

Authors agree that the best approach would be an individualized one, with careful decision regarding the invasiveness and optimal time of therapy. Despite the increasing awareness of the risks associated with this condition, the mortality rate of Bouveret’s syndrome remains high even after therapy, and is mostly attributed to the comorbidities and advanced age of the affected patients. Non-specific symptoms and signs, tricky therapeutic approaches with new evidence of clinical and histological variations are enough to make of Bouveret’s syndrome a real medical and surgical challenge.
